# Direct cost of health care for individuals with community associated *Clostridium difficile* infections: A population-based cohort study

**DOI:** 10.1371/journal.pone.0224609

**Published:** 2019-11-08

**Authors:** Harminder Singh, Zoann Nugent, A Walkty, B Nancy Yu, Lisa M. Lix, Laura E. Targownik, Charles N. Bernstein, Julia Witt

**Affiliations:** 1 University of Manitoba IBD Clinical and Research Center, Winnipeg, Manitoba, Canada; 2 Department of Internal Medicine, University of Manitoba, Max Rady College of Medicine, Winnipeg, Manitoba, Canada; 3 CancerCare Manitoba, Research Institute, Winnipeg, Manitoba, Canada; 4 Department of Community Health Sciences, University of Manitoba, Winnipeg, Manitoba, Canada; 5 Public Health Branch, Manitoba Health, Seniors and Active Living, Winnipeg, Manitoba, Canada; 6 Department of Economics, University of Manitoba, Winnipeg, Manitoba, Canada; Stanford University, UNITED STATES

## Abstract

**Background:**

Even though the incidence of community-acquired *Clostridium difficile* infection (CDI) is reported to be increasing, few studies have reported on the healthcare costs of community-acquired CDI. We estimated cost of care for individuals with community-associated CDI and compared with that for matched controls without CDI in the time period of six months before to one year after CDI.

**Methods:**

All individuals in the province of Manitoba, diagnosed with CDI between July 2005 and March 2015 were matched up to 4 individuals without CDI. Health care utilization and direct costs resulting from hospitalizations, physician reimbursement claims and prescriptions were determined from the population based provincial databases. Quantile regressions were performed to determine predictors of cost of individuals with community associated CDI.

**Results:**

Of all CDIs, 30–40% in each period of the study had community-associated CDI; of which 12% were recurrent CDIs. The incremental median and 90^th^ percentile cost of care for individuals with community-associated CDI was $800 and $16,000 respectively in the six months after CDI diagnosis. After adjustment for age, co-morbidities, sex, socioeconomic status and magnitude of health care utilization prior to CDI, the median incremental cost for recurrent CDI was $1,812 and that for a subsequent episode of CDI was $3,139 compared to those with a single community-associated CDI episode. The median cost for a prescription of Vancomycin was $316 (IQR 209–489).

**Conclusions:**

Health care costs of an episode of community-associated CDI have been much more than the cost of antibiotic treatment. Our study provides population-based data for formal cost effectiveness analysis for use of newer treatments for community-associated CDI.

## Introduction

*Clostridium difficile* (CD) is a Gram-positive anaerobic spore forming bacterium that produces toxins and can lead to clinically significant diarrhea and substantial morbidity and mortality[1]. Over the last decade several hospital based outbreaks with a newer more toxic strain have been reported in North America[2, 3] Moreover, an increasing occurrence of CD infections (CDI) has been reported over the last decade[4], including in ambulatory care settings[2]. The increasing incidence and associated morbidity and mortality have created an impetus to develop newer therapeutic approaches[1, 5]. While these newer agents are more expensive than traditional antibiotics, downstream health care savings could be substantive if the improved effectiveness of these regimens leads to rapid cure and/or a lower incidence of recurrent CDI and less associated morbidity. It is necessary to understand the traditional cost of initial and recurrent CDI before undertaking evaluations of the cost-effectiveness of novel, more expensive therapies.

Although up to 40% of CDI have been reported to be community-associated[6], few studies have evaluated health care costs associated specifically with community-associated CDI (ca-CDI)[7].

Recent guidelines, based on efficacy data, now recommend vancomycin or fidaxomicin rather than the traditionally used metronidazole, including for ca- CDI[8]. However, cost and utilization analyses were not addressed in these guidelines. Cost analyses from the perspective of payors of direct costs are essential to determine the cost-effectiveness of the recommendations.

Much of the information on the costs of CDI in North America has been reported by identifying CDI from International Classification of Diseases (ICD) codes for CDI in secondarily collected administrative health records or from single center studies[9–11]. However, the use of ICD codes to identify CDI has been reported to be inaccurate in several studies, overestimating the number of CDI cases relative to use of the toxin assay[12]; limited positive predictive value (71.6%; 95% Confidence Interval (CI): 62.1–86.6%)[13] and missing up to 30% of hospitalized CDI cases[14]. Reported per-patient costs of CDI vary by 2 orders of magnitude among different hospitals[15], which maybe at least partly due to the use of non-validated administrative data definitions of CDI to identify individuals with CDI. In addition, use of hospital discharge data does not allow assessment of CDI in the outpatient setting or determination of the precise date when a CDI infection was diagnosed.

In Manitoba, reporting of all positive CD assays was legally mandated since 2005[16] (https://www.gov.mb.ca/health/publichealth/act.html), which has allowed us to create a population-based comprehensive dataset of CDI cases and evaluate CDI epidemiology and costs of care on a population basis. In this manuscript, we report the estimated cost of care of individuals with ca-CDI compared with matched controls without CDI.

## Methods

### Data sources

Manitoba is a central Canadian province with a relatively stable population (population in 2014: 1.3 million)[17]. Manitoba Health is the provincial agency which oversees the delivery of universal health care in the province. Only members of the Canadian armed forces, national police and inmates of federal penitentiaries are excluded and covered by the federal government. Manitoba Health maintains several electronic administrative healthcare databases to monitor the services delivered and for re-imbursement to health care providers for the services rendered[18–21]. Up to 16 ICD-9 (prior to 2004) or 25 ICD-10 (since 2004) codes are recorded for each inpatient hospital stay, whereas each outpatient physician visit is coded with a single ICD-9 code. Records of all outpatient prescriptions dispensed since 1995 are recorded in the Drug Programs Information Network (DPIN) Database.

The Manitoba Health Public Health Branch Epidemiology and Surveillance Unit maintains a population-based CDI dataset since 2005, developed from the legally mandated universal reporting of documented CDI cases in the province to the unit. Diagnostic testing for CDI is performed in six public laboratories, of which 3 perform approximately 84% of the testing. Only loose stool, which takes shape of its container has been tested by the laboratories, thereby minimizing the detection of asymptomatic carriers (estimated to be 2–7% in the population[22, 23]).

Between 2005 and May 2013, the laboratories in Manitoba performed immunoassays for the glutamate dehydrogenase (GD) antigen and *C*. *difficile* toxins A and B, followed by the cytopathic effect (CPE) assay (using viable human fibroblasts) and/or culture for discordant results (i.e., GD antigen positive but C. difficile toxin A & B immunoassay negative)[24, 25]. The predominant assays used during the study years were the C. Diff Quik Chek test (Techlab Inc., Blacksburg, VA) for the GD antigen, and the Tox A/B Quik Chek test (Techlab Inc., Blacksburg, VA) for C. difficile toxins A and B. Since May 2013, the Nucleic Acid Amplification Test (NAAT, using a Health Canada cleared nucleic acid amplification assay for the detection of a segment of *Clostridium difficile* DNA known to be present in all known toxigenic strains of *C*. *difficile*, including A-B+ toxin types) is used for confirmation of CDI[25].

Since 1984, all individuals in the province have been assigned a unique personal health identification number (PHIN). Scrambled anonymized PHINs were used to link the data in above databases for the current study.

### CDI cases and controls

All individuals in the province, diagnosed with one or more episodes of CDI between July 2005 and March 2015 were matched with up to 4 individuals without CDI based on sex, age (± 5 years), area of residence (first 3 digits of the postal code) and duration of coverage with Manitoba Health prior to the CDI onset (approximated by the date of stool specimen collection; defined as the index date in the study) of each episode. Individuals with ca-CDI and their controls were included in the analysis for this report. Those younger than 18 years of age or residents of long-stay care facilities on the index date were excluded as we did not have access to person-level costing data from long-stay care facilities. All individuals were followed from one year prior to the index date to death, out-migration from the province, admission to a long-stay care facility or end of the study time period (March 2015).

Previously recommended and used definitions (S1 Table) for CDI[16, 26] were used in the study to determine the site of acquiring CDI and whether it was incident or recurrent, if it was severe and whether it was the first or a subsequent infection. Second/later episodes were defined as subsequent infections not meeting the definition of recurrent infections.

### Costs

Direct costs resulting from hospitalizations, physician reimbursement claims and prescriptions were determined from the Manitoba Health databases. Inpatient hospitalization costs were calculated using data in each record from the Canadian Institute for Health Information (CIHI). Specifically, using a case mix methodology, CIHI groups together patients with similar clinical and resource-utilization characteristics, and uses this information to calculate a resource intensity weight (RIW) for each episode of care. This RIW reflects in hospital resource utilization, and is higher for more complex patients. An “average” resource-use patient would have a RIW = 1. Additionally, CIHI uses annual hospital budgets to calculate province-specific costs associated with an “average” hospital stay. This Cost per Standard Hospital Stay (CSHS) reflects the cost of one inpatient hospital stay with a RIW = 1 in a particular year. Thus, to derive inpatient hospitalization costs for each episode, RIW is multiplied by CSHS for each record in the hospital data. Case Mix Groups (CMGs), modeled after the American Diagnosis Related Groups (DRG's), represent a Canadian patient classification system used to group and describe types of inpatients with similar clinical characteristics and resource utilization discharged from acute-care hospitals.

RIW’s and financial data may change from one year to another due to changes in methodology or adjustments. However, CIHI provides grouper data for inpatient hospitalizations to make costing data over time periods comparable. The costs of outpatient (day) procedures were included, calculated using annual RIW and CSHS values. Physician visits costs were reimbursements for fee-for-service claims as listed in the MH provider re-imbursement claims dataset, and were applied for both inpatient and outpatient physician services. Prescription costs reflect charges paid by patients, the province and/or the insurance companies and include dispensing fee and the cost of the drugs. Although, inpatient dispensations from hospital pharmacies are not captured in DPIN, inpatient pharmacy costs are included in CSHS. All costs were adjusted to 2016 Canadian dollars using the Consumer Price Index (CPI).

### Statistical analysis

Costs were calculated for 6-month time periods, starting from one year preceding each ca-CDI episode. We included cost estimates prior to the ca-CDI episode as many individuals with CDI have predisposing comorbid conditions, with associated higher costs of healthcare, irrespective of occurrence of CDI. Since migration and death reduce the time period of observation and therefore potentially reduce costs, all individuals had to be registered during the entire six month time periods that they contributed the data. Quantile regression models were selected because the study data included some very high cost patients leading to skewed distributions. The percentiles selected for modeling were the 50^th^ (median), 75^th^ and 90^th^. Covariates in the models included, in addition to group membership (i.e., case, control) age, sex, diabetes, dialysis, Charlson co-morbidity index (CCI) score, the socio-economic factor index (SEFI), and number of physician visits (categorised into quartiles) in the year preceding the time period evaluated. The CCI score (categorised as 0,1, 2, 3 or more) was calculated from diagnoses listed in hospitalizations records and physician claims in the year preceding the evaluated time period; diabetes and dialysis were not included in calculation of CCI score; rather, they were entered as separate covariates because they are relatively common diseases associated with CDI[27–29]. The SEFI is a validated measure of socio-economic status and is based on several neighborhood level measures of wealth[30]. SEFI scores can range from minus 3 to plus 7, with higher scores indicating greater deprivation. SEFI was included in the models as a continuous variable. Individuals were censored at the beginning of the time period in which they entered long-stay care, died or moved out of province.

Data management and analyses were performed using SAS version 9.4 (SAS Institute, Cary, NC). This study was approved by the University of Manitoba’s Health Research Ethics Board and the Health Information and Privacy Committee of Manitoba Health.

## Results

There were 6,234 individuals aged 18 or older, who experienced 8,471 episodes of CDI between July 1, 2005 and March 31, 2015 in the province. 372 cases with CDI and 1188 controls without CDI were excluded because they were residents of long-stay care facilities prior to CDI diagnosis. After excluding CDI cases with no remaining matching controls, there were 5,852 cases experiencing their first recorded CDI and their 22,041 matching controls.

30–40% of individuals with CDI in each of the follow-up time periods had ca-CDI and were included in the analysis for this report. Their characteristics are provided in Table 1. Approximately 12% of ca-CDI episodes were recurrent CDIs. Individuals with ca-CDI were more likely to have diabetes, receive dialysis or have higher CCI score (i.e. co-morbidity burden) than their controls (Table 1).

**Table 1:** Characteristics of CDI cases and controls without community associated CDI by time period of follow-up before and after CDI diagnosis.

Time	1 year—6 months prior		6 months—1 day prior		CDI—6 months post		6 months—1 year post	
	Case	Control	Case	Control	Case	Control	Case	Control
N	1,887	7,307	1,887	7,307	1,637	6,321	1,503	5,740
Male (%)	37.5	37.7	37.5	37.7	37.5	37.4	37.8	37.6
Dialysis (%)	1.8	0.1	2.0	0.1	1.4	0.1	1.3	0.1
Diabetes diagnosis (%)	15.0	12.4	15.6	13.0	14.4	12.7	14.5	13.0
Charlson Co-morbidity Index score (without Diabetes or Renal failure) (%)								
0	68.3	81.9	66.7	80.9	68.3	81.2	65.8	81.2
1	17.6	11.2	17.4	11.9	16.8	11.8	16.8	11.5
2	9.2	5.1	10.3	5.3	9.7	5.3	10.5	5.4
3+	4.9	1.8	5.6	2.0	5.2	1.7	6.9	1.9
Age (years)								
Median	56	55	56	55	55	54	56	55
Lower Quartile	41	40	41	41	40	40	41	41
Upper Quartile	70	69	71	69	68	67	69	67
Ambulatory Care Contacts in the year prior to the evaluated time period (n)								
Median	10	6	10	6	12	6	15	6
Lower Quartile	5	3	5	3	6	2	9	3
Upper Quartile	19	12	19	12	20	12	24	12
SEFI Socio-economic Measure								
Median	-0.18	-0.18	-0.18	-0.18	-0.21	-0.19	-0.21	-0.19
Lower Quartile	-0.70	-0.69	-0.70	-0.69	-0.72	-0.70	-0.72	-0.71
Upper Quartile	0.27	0.28	0.27	0.28	0.26	0.27	0.26	0.27
Recurrence (% of all CDI cases)								
Yes	12.7		12.7		12.2		11.9	
No, but later infection (i.e. 2 or more episodes)	5.9		5.9		5.8		5.7	
No (incident only/ single episode)	81.4		81.4		82.0		82.4	

The median cost of care for individuals with ca- CDI was higher than the median cost for their controls in all of the time periods, including even 6 months to a year before CDI diagnosis; the incremental cost for individuals with ca-CDI compared to those without CDI was highest in the six months after ca-CDI diagnosis—an increase of median cost of $800 and 90^th^ percentile cost of approximately $16,000 among those with ca-CDI as compared to the six month cost before ca-CDI (Table 2). The costs remained stable among those without CDI.

**Table 2:** Cost of healthcare for CDI cases and controls without community-associated CDI by time period of follow-up before and after CDI diagnosis (2016 constant Canadian dollars).

	Number of Individuals	Total annual cost	Median	75^th^ percentile	90^th^ percentile
12–6 m prior to index date					
CDI cases	1887	15,961,986	682	2088	8110
Controls	7307	19,503,288	317	863	2156
Incremental adjusted CDI Cost (from multivariable model)			137	312	1473
6 m– 1 day prior to index date					
CDI cases	1887	13,596,512	933	2566	8554
Controls	7307	21,062,756	319	926	2339
Incremental adjusted CDI Cost (from multivariable model)			330	610	1895
CDI– 6 m post CDI					
CDI cases	1637	26,905,230	1561	6107	16358
Controls	6321	18,478,716	313	875	2211
Incremental adjusted CDI Cost (from multivariable model)			734	3916	8186
6–12 m post CDI					
CDI cases	1503	11,645,036	722	2133	8009
Controls	5740	14,974,800	307	856	2180
Incremental adjusted Cost (from multivariable model)			68	192	1397

Incremental adjusted CDI Cost from multivariate model, adjusted for differences between those with and without CDI, such as presence of comorbidities, which influence costs.

In the multivariable quantile regression analysis, the incremental costs in the six months after ca-CDI diagnosis for the individuals with ca-CDI as compared to those without CDI had an estimated median of $734, 75^th^ percentile of $3,916 and 90^th^ percentile of $8,186, which were approximately $400, $3300 and $6000 more than in the six months before ca-CDI (Table 3).

**Table 3:** Estimates from multivariable quantile regression models for additional cost of healthcare (in 2016 Canadian dollars) for individuals with community-associated CDI by time period of follow-up before and after CDI diagnosis.

	Median (95% CI) costs				75th percentile (95% CI) costs				90th percentile (95% CI) costs			
	Before CDI		After CDI		Before CDI		After CDI		Before CDI		After CDI	
	1y – 6m	6m – 1d	diagnosis– 6m	6m – 1y	1y – 6m	6m – 1d	CDI– 6m	6m – 1y	1y – 6m	6m – 1d	CDI– 6m	6m – 1y
CDI vs without CDI	137	330	734	68	312	610	3916	192	1473	1895	8186	1397
	(124, 150)	(317, 344)	(717, 752)	(47, 88)	(276, 348)	(573, 648)	(3875, 3957)	(149, 236)	(1355, 1591)	(1761, 2029)	(8072, 8300)	(1260, 1535)

Multivariable regression analysis was performed for ca-CDI cases alone, with categorisation of ca-CDI as single episode, with recurrent episode and second/later episode and incremental costs were compared to those with a single episode alone. In this analysis, although the incremental median costs in the first six months after the ca-CDI diagnosis were higher for second/later episode than recurrent episode, ($3,139 and $1,812 respectively), the 75^th^ and 90^th^ percentile costs were higher for those with recurrent CDIs (75^th^ percentile recurrent $7,086, later episode $5,113; 90^th^ percentile recurrent $25, 256, later episode $20,013)(Table 4).

**Table 4:** Multivariable quantile regression models for potential predictors of cost of care (in 2016 Canadian dollars) among individuals with community-associated CDI by time period of follow up (before and after initial CDI) *.

		Median (95% CI) costs				75th percentile (95% CI) costs				90th percentile (95% CI) costs			
		Before CDI		After CDI		Before CDI		After CDI		Before CDI		After CDI	
		1y – 6m	6m – 1d	CDI– 6m	6m – 1y	1y – 6m	6m – 1d	CDI– 6m	6m – 1y	1y – 6m	6m – 1d	CDI– 6m	6m – 1y
Intercept		153	296	491	274	344	699	1463	378	891	1137	5008	962
		(60, 246)	(166, 425)	(153, 830)	(89, 459)	(104, 584)	(385, 1013)	(107, 2819)	(-100, 856)	(93, 1689)	(-158, 2431)	(994, 9022)	(-1423, 3347)
Type of CDI	Recurrent vs Single Episode	28	157	1812	21	107	4	7086	-43	1386	380	25256	628
		(-53, 109)	(47, 267)	(1559, 2064)	(-90, 132)	(-102, 316)	(-263, 270)	(6074, 8098)	(-330, 243)	(691, 2080)	(-718, 1478)	(22261, 28252)	(-800, 2057)
	Second/later vs Single episode	172	186	3139	329	508	1348	5113	1312	5436	3574	20013	12156
		(59, 286)	(32, 339)	(2782, 3496)	(172, 486)	(215, 801)	(975, 1721)	(3683, 6542)	(908, 1717)	(4462, 6410)	(2037, 5110)	(15782, 24244)	(10139, 14173)
Male vs Female		-3	31	28	-94	108	141	1061	-56	810	1329	3975	604
		(-58, 52)	(-44, 105)	(-142, 198)	(-167, -21)	(-33, 250)	(-40, 322)	(381, 1742)	(-244, 133)	(338, 1282)	(584, 2074)	(1961, 5989)	(-336, 1544)
Diabetes Diagnosis		717	904	1245	634	1962	1669	1070	925	6467	3445	-1317	3929
		(638, 796)	(799, 1009)	(994, 1495)	(527, 740)	(1759, 2165)	(1414, 1925)	(66, 2074)	(651, 1198)	(5792, 7143)	(2392, 4498)	(-4289, 1655)	(2563, 5295)
Dialysis		15044	5752	21650	5566	48162	14550	60410	38966	108427	29536	197689	63974
		(14840, 15247)	(5488, 6017)	(20935, 22365)	(5244, 5889)	(47638, 48685)	(13908, 15193)	(57545, 63275)	(38135, 39798)	(106684, 110169)	(26889, 32183)	(189209, 206170)	(59826, 68123)
SEFI		-7	-15	59	0	21	71	227	26	153	34	818	114
		(-38, 24)	(-56, 27)	(-35, 153)	(-40, 40)	(-57, 100)	(-30, 171)	(-149, 602)	(-79, 130)	(-109, 415)	(-381, 449)	(-295, 1930)	(-405, 634)
Age (yrs) (reference category 60–69)	18–39	-110	-121	-318	-180	-161	-260	-679	-127	-528	-311	-1119	-457
		(-197, -23)	(-239, -3)	(-582, -55)	(-292, -67)	(-385, 63)	(-546, 26)	(-1735, 378)	(-417, 163)	(-1273, 217)	(-1488, 866)	(-4246, 2008)	(-1904, 989)
	40–49	-83	-108	-262	-136	-195	-255	-781	-62	-355	-139	-1510	-492
		(-178, 13)	(-238, 21)	(-553, 29)	(-259, -13)	(-440, 49)	(-570, 59)	(-1949, 386)	(-379, 255)	(-1170, 460)	(-1436, 1159)	(-4965, 1945)	(-2074, 1090)
	50–59	21	-32	-243	41	-8	-17	-665	189	-339	159	2326	655
		(-68, 110)	(-152, 87)	(-508, 22)	(-73, 155)	(-238, 221)	(-307, 273)	(-1726, 397)	(-105, 482)	(-1102, 424)	(-1037, 1355)	(-816, 5468)	(-810, 2120)
	70–79	231	332	979	33	183	189	2794	444	985	127	6712	429
		(135, 326)	(203, 462)	(684, 1274)	(-91, 158)	(-62, 429)	(-125, 504)	(1613, 3975)	(122, 765)	(168, 1802)	(-1170, 1424)	(3216, 10207)	(-1174, 2032)
	80+	487	269	2483	174	813	456	6454	222	985	2785	13278	1665
		(386, 588)	(136, 401)	(2155, 2811)	(36, 313)	(553, 1073)	(133, 778)	(5140, 7768)	(-135, 579)	(121, 1850)	(1455, 4114)	(9389, 17168)	(-117, 3446)
CCI score (reference: 0)	3+	1592	2193	1817	1591	4852	5449	5590	5205	25994	6111	3139	5182
		(1460, 1723)	(2024, 2361)	(1424, 2209)	(1441, 1740)	(4514, 5191)	(5040, 5858)	(4017, 7163)	(4820, 5590)	(24867, 27121)	(4427, 7795)	(-1517, 7795)	(3261, 7103)
	2	835	805	1191	782	3189	3369	6223	1829	12390	8685	11948	5482
		(736, 934)	(675, 935)	(894, 1488)	(659, 905)	(2934, 3445)	(3054, 3685)	(5033, 7413)	(1512, 2147)	(11540, 13240)	(7384, 9985)	(8425, 15470)	(3897, 7068)
	1	342	142	321	176	805	236	788	169	4116	1743	3598	195
		(268, 417)	(40, 244)	(91, 552)	(78, 275)	(613, 997)	(-10, 483)	(-135, 1712)	(-85, 422)	(3477, 4755)	(726, 2759)	(863, 6332)	(-1071, 1461)
Physician Usage (by quartile of use) (reference: Lowest Quartile)	Highest	1081	1349	1529	736	2351	2759	4062	2455	5704	8496	6515	6330
		(993, 1168)	(1229, 1469)	(1218, 1840)	(561, 911)	(2126, 2575)	(2468, 3050)	(2817, 5307)	(2004, 2906)	(4956, 6452)	(7297, 9695)	(2830, 10201)	(4078, 8581)
	Third	349	332	391	191	635	621	851	391	1318	1655	-38	638
		(264, 433)	(215, 449)	(87, 695)	(16, 366)	(418, 851)	(337, 905)	(-367, 2068)	(-60, 842)	(597, 2039)	(486, 2825)	(-3642, 3566)	(-1610, 2887)
	Second	117	148	118	64	253	176	256	121	393	198	-1368	270
		(33, 200)	(31, 264)	(-197, 433)	(-121, 249)	(37, 468)	(-107, 459)	(-1006, 1518)	(-357, 598)	(-325, 1111)	(-969, 1365)	(-5103, 2368)	(-2113, 2654)

* Each time period analysis was limited to those surviving to the end of the period.

Intercepts represent the cost when all variables are set to reference values for example, the intercept provides the median cost for a 60–69 year old female subject without, diabetes or dialysis, who had median SEFI score and no comorbidities contributing to Charlson comorbidity score and low previous ambulatory care use.

The 75^th^ and 90^th^ percentile regression analyses suggest the incremental costs for the most expensive 25 percent of individuals with recurrent ca-CDI are $7,086 per person and that for the most expensive 10 percent of individuals with recurrent ca-CDI are $ 20,013 per person in the first six months after the initial ca-CDI.

Increasing age, male sex, higher CCI score (co-morbidities), diabetes, dialysis and prior ambulatory care use were associated with increased cost of care among those with ca-CDI, both before and after the ca-CDI index date; the incremental costs with presence of each these factors were much higher in the six months after ca-CDI diagnosis.

**Fig 1:**
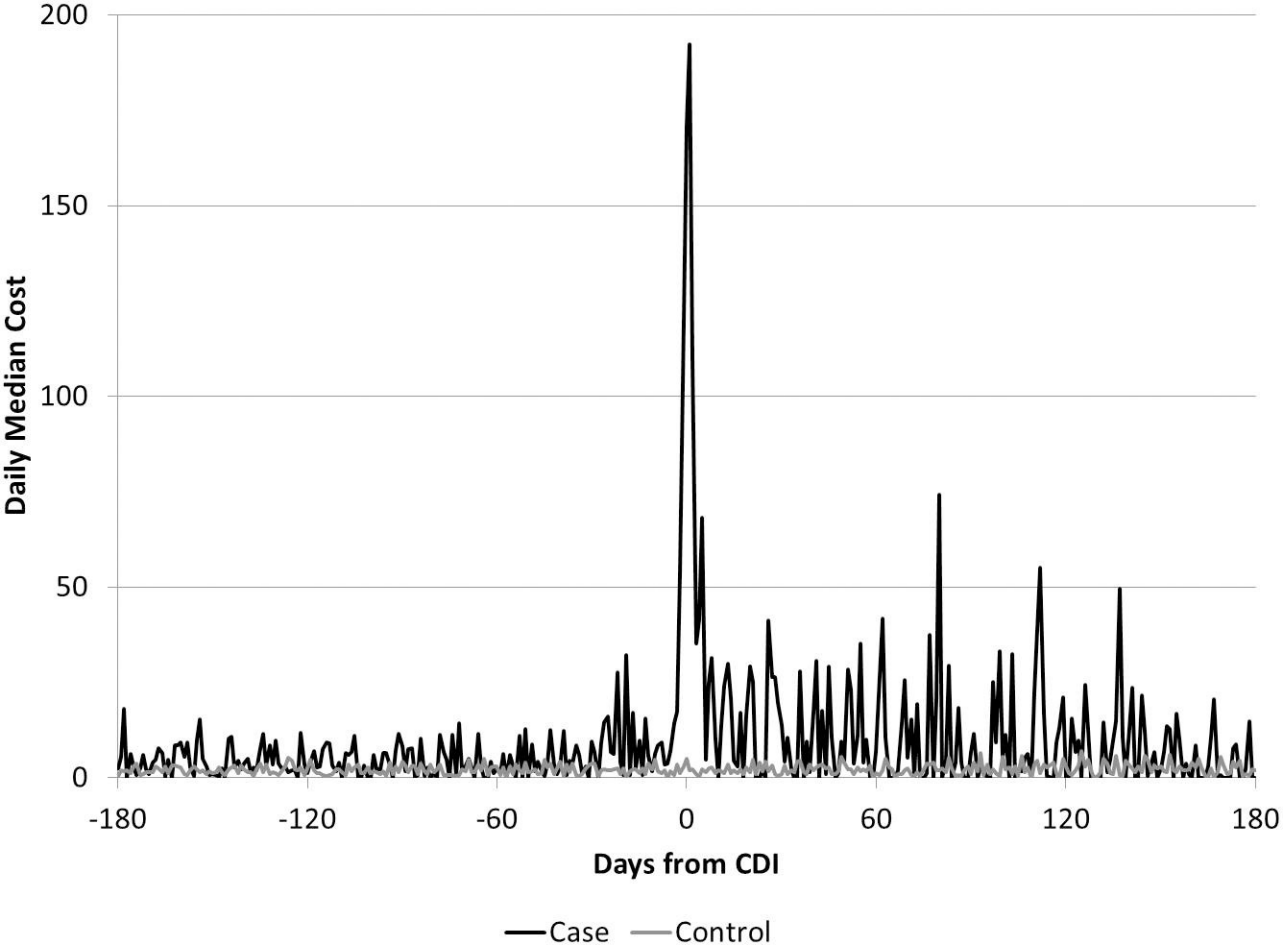
Daily median costs for CDI cases and controls without community-associated CDI, over six months before and after index date.

**Fig 2:**
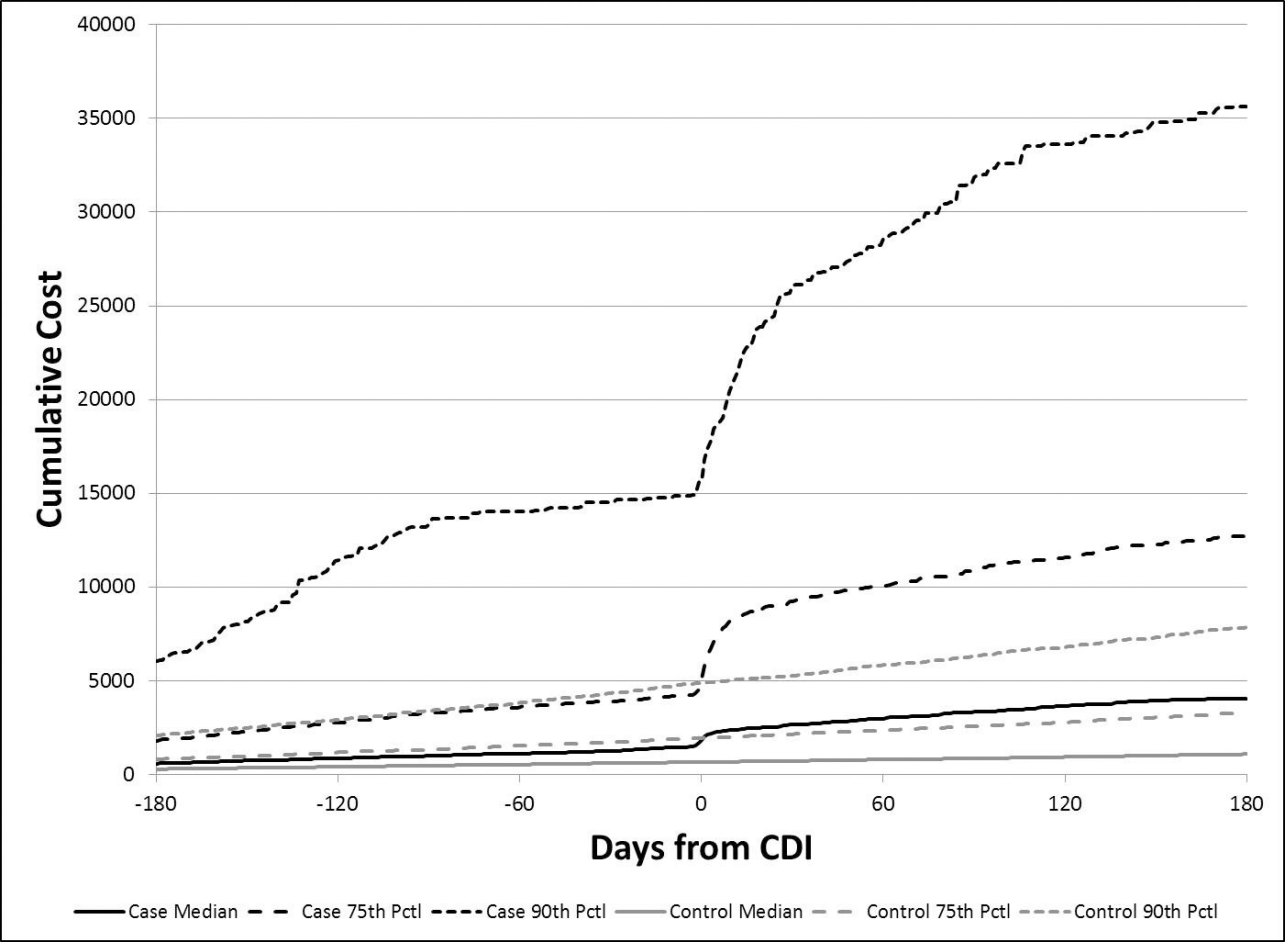
Cumulative median, 75^th^ percentile, and 90^th^ percentile costs for community-associated CDI cases and controls without CDI.

**Fig 3:**
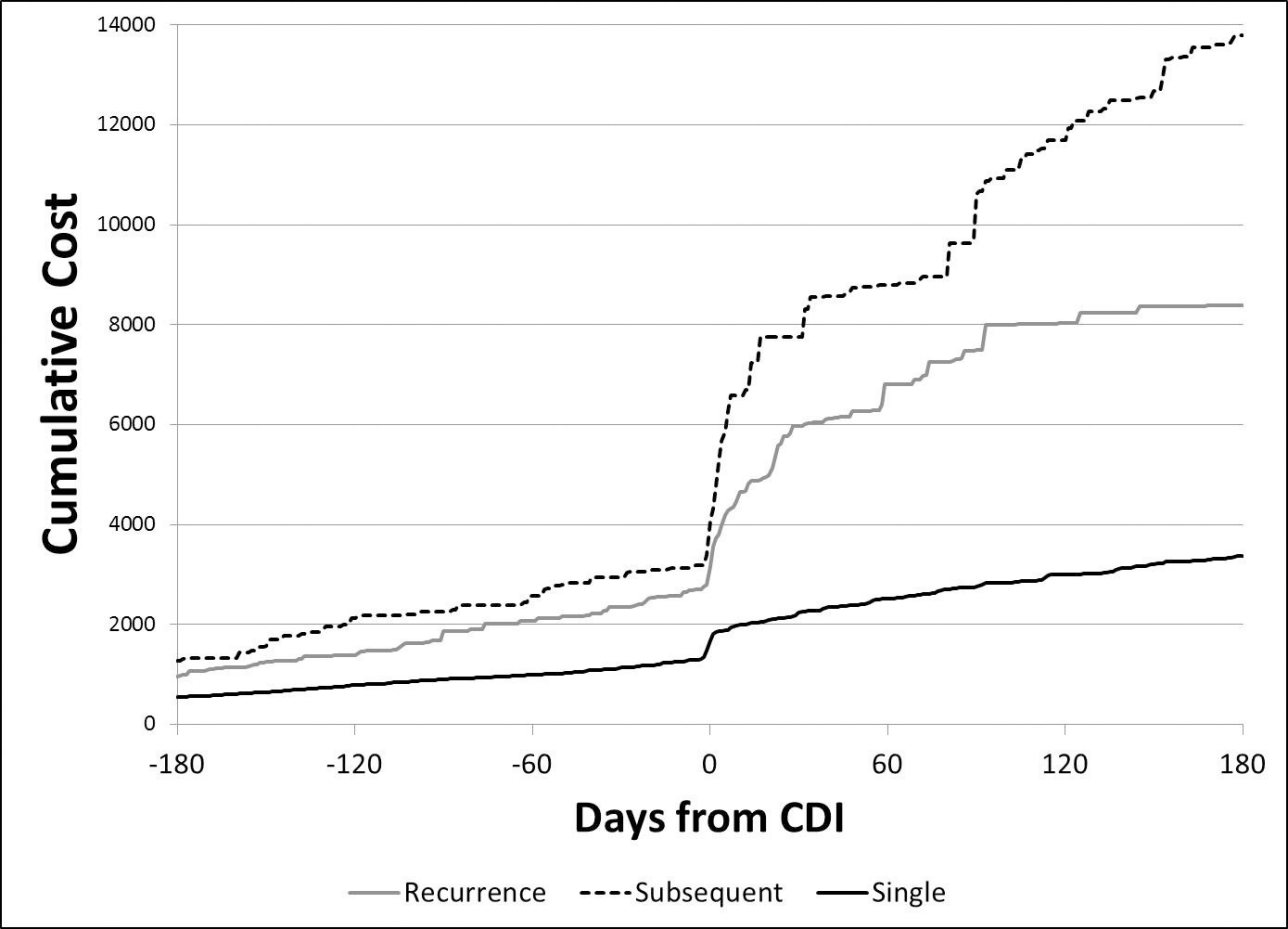
Cumulative median costs for those with single episode of community-associated CDI, recurrent and one or more subsequent CDI episodes.

The plot of daily median costs among those with and without ca-CDI (Fig 1) suggests that although the cost differential was highest soon after diagnosis, it continued for several months. The cumulative costs are displayed in Figs 2 and 3 and suggest cumulative median costs among those with subsequent CDI episodes were higher than among those with recurrent episodes (Fig 3)

The median cost of all antibiotic prescriptions for those with first episode was $8 (mean cost $89) and for that with recurrent/second or later episode of CDI was $85 (mean cost $474). The median cost for a prescription of vancomycin was $316 (Interquartile range (IQR) $209-$489) and for metronidazole it was $4 (IQR $3-$5).

## Discussion

In this province wide population-based study, we are reporting markedly incremental costs of those with recurrent and repeated CDIs after an initial ca-CDI as compared to those with single ca-CDI episode. We report incremental costs of care of individuals with ca-CDI even before ca-CDI diagnosis, predictors of increased cost among those with ca-CDI and that ca-CDI increases cost of care much more in older individuals. These data would be valuable for cost-effectiveness studies evaluating benefits of newer therapies to treat ca-CDI and prevent recurrent and repeat CDIs after the initial ca-CDI. These data also provide economic rationale for prevention of ca-CDI from the perspective of payors of health care services.

Nana et al. estimated median attributable six months costs of ca-CDI of $7,394 (2014 Canadian dollars), using administrative health care data in Ontario for CDIs between 2003 and 2010[31]. However, they were not able to include individuals who did not visit Emergency departments and/or were not admitted (i.e. who only had physician office visits for CDI) and hence likely reported costs of the more severe ca-CDI cases i.e. costs of ca-CDI leading to hospitalization. They were also limited by the limitations of using ICD codes to identify CDI. We are not aware of other prior studies evaluating incremental cost of care of individuals with ca-CDI. A systematic review published in 2014, estimated CDI attributable costs expressed in US dollars, as $6,774-$10,212 for CDI requiring admission and $2,992-$29,000 for hospital-acquired CDI[32]. Other estimates of the per-patient cost of CDI in the US have varied markedly between individual hospitals[15]. Levy et al. (2015) estimated the burden of CDI in Canada, including direct and indirect (productivity loss) costs using projections of CDI and costs from the literature. At the episode level, they estimated that the incremental hospitalization cost, excluding pharmacotherapy, was $11,928 for each initial infection, and $15,330 for each recurrent infection (in 2012 Canadian dollars)[33]. Our estimates are lower, likely as we included milder cases and individuals who never presented to the EDs or hospitals. Even then, as noted in Table 2, there are substantial cumulative total costs associated with ca- CDI.

Our study suggests that even when the analysis is adjusted for several baseline characteristic differences among those predisposed to develop ca-CDI and those who do not develop CDI, the cost of care is higher even before ca-CDI episode. We believe, this differential cost at baseline should be considered in any cost effectiveness study evaluating the effect of different treatments for CDI and not merely limit to matching those with and without CDI as that may not adjust for underlying cost differential among people predisposed to CDI vs not.

Our study found pharmaceutical costs were a small component of increased costs among those with recurrent/subsequent episode of CDI. Therefore, the increased costs are more likely due to increase in other health care use. The additional mean pharmaceutical costs were similar to that of cost of a prescription for vancomycin, which was approximately $400. This alone would support the use of vancomycin as the preferred agent of recurrent/subsequent episode of ca-CDI, with no net increase in pharmaceutical costs.

There are no data on cost-effectiveness of different strategies for ca-CDI; our study provides cost data using traditional approach for such analyses. While similar recurrence rates have been reported with use of vancomycin and metronidazole[34], because of its lower efficacy for clinical response, metronidazole is no longer recommended and instead vancomycin or fidaxomicin are recommended as first line treatments for all cases of CDI[8]. Fidaxomicin has been reported to be noninferior to vancomycin for clinical cure (fidaxomicin, 88% vs vancomycin, 86%) and associated with much lower recurrence rate(15.4% vs. 25.3%, P = 0.005)[35]. Even though fidaxomicin is much more expensive than vancomcycin (current (April 2019) mean cost in Manitoba for the recommended 10 day prescription for fidaxomicin is $2016.16, including dispensing fee), fidaxomicin was a more cost-effective therapy than metronidazole or vancomycin for initial CDI, in 2 of 3 studies identified in a recent systematic review[36]. However, there are no cost-effectiveness data for ca-CDI.

We are reporting increasing age, male sex, higher CCI score (co-morbidities), diabetes, dialysis and higher prior ambulatory care use are associated with increased incremental cost of care (median as well as higher percentiles) among those with ca-CDI. Purely on economic basis, therefore, individuals with these factors should particularly be considered for use of therapies with higher efficacy for clinical cure and/or lower recurrent episodes.

Our results should be viewed in the context of strengths and limitations of our study. We are reporting direct costs of care in a population-based setting of ca-CDI. There are many studies on CDI costing, but health care costs of this sub-group of individuals with CDI has not been previously extensively investigated, likely because of limited truly population-based datasets of ca-CDI We were able to identify CDI, using a laboratory confirmed cases dataset. We are providing costs by initial and subsequent CDI and predictors of cost in the population setting. However, in the time period of this study, metronidazole was the standard of care for first episode of mild CDI, which is no longer recommended. Our study does suggest that health care costs of ca-CDI were much more than the cost of an average prescription of vancomycin and therefore higher efficacy of vancomycin could potentially lower overall costs of ca-CDI by reducing the subsequent health care utilization—this will need to be analyzed in cost effectiveness studies. We included dialysis patients in the ca-CDI category, although some dialysis units are located in the hospitals; however, most are in ambulatory care centers and the recommended definition in the time period of the study included them in the community associated category.

In conclusion, we report costs for care of individuals with ca- CDI in a province wide setting that highlight the need of prevention of ca-CDI as well as therapies which can lead to rapid cure and prevent recurrence in community setting also of CDI. Health care costs of an episode of ca- CDI are much more than the cost of antibiotic treatment. Our study provides population-based data for use in future studies that evaluate the cost-effectiveness of various CDI therapies for ca-CDI.

## Supporting information

S1 Table Definitions of clostridium difficile infections. (DOCX)
