# Supplementary material for: Direct cost of health care for individuals with community associated Clostridium difficile infections: A population-based cohort study
Source: PLoS One. 2019 Nov 8;14(11):e0224609. doi: 10.1371/journal.pone.0224609 (PMC6839863; doi:10.1371/journal.pone.0224609)
Supplement: S1 Table — (DOCX) [file pone.0224609.s001.docx]

**Supplementary table S1: Definitions of Clostridium Difficile Infections**[**^20^**](#_ENREF_20)**^,^** [**^21^**](#_ENREF_21)

| **Type** | **Definition** |
| --- | --- |
| Health care facility (HCF) onset, HCF-associated CDI | Positive specimen collected more than 48 hours after admission to a HCF and before discharge |
| Community-onset, HCF-associated CDI | Positive specimen collected while in the community or within 48 hours after admission to a HCF, provided the individual had been discharged from a HCF less than 4 weeks prior |
| Community-associated CDI | Positive specimen collected while in the community or within 48 hours after admission to a HCF, provided the individual either had never been in a HCF or was discharged from a HCF more than 12 weeks prior to CDI onset. |
| Indeterminate CDI | Does not fit in the previous classifications e.g. Positive specimen collected in the community but the individual was discharged from the same or another HCF 4-12 weeks prior to specimen collection. |
| Incident CDI | Positive specimen result 8 or more weeks after a previous positive result or no prior positive result in the individual |
| Recurrent CDI | Positive specimen result within 2–8 weeks of the last positive result. A positive test result within 2 weeks of the previous positive result is considered a confirmation of the initial positive result. |
| Severe CDI | Within 30 days of a positive specimen: admission to an ICU, colectomy or death |
